# Female cancer incidence before and after diagnosis of primary Sjögren's disease: A retrospective cohort study

**DOI:** 10.1016/j.jtauto.2026.100352

**Published:** 2026-01-21

**Authors:** Bitte Sjöström, Anders Bredberg, Peter Olsson, Susann Ullén, Johannes H. van der Stoep, Gunnel Henriksson

**Affiliations:** a Department of Laboratory Medicine, Lund University, Lund, Sweden; b Department of Medical Microbiology, Innlandet Hospital Trust, Lillehammer, Norway; c Department of Clinical Sciences Malmö, Rheumatology, Lund University, Malmö, Sweden; d Department of Rheumatology, Skåne University Hospital, Malmö, Sweden; e Clinical Studies Sweden – Forum South, Skåne University Hospital, Lund, Sweden; f Department of Pathology, Innlandet Hospital Trust, Lillehammer, Norway; g Department of Clinical Microbiology, Infection Prevention and Control, Skåne University Hospital, Lund, Sweden

**Keywords:** Sjögren's disease, Sjögren's syndrome, Breast cancer, Gynecological cancer, Female cancer, Autoantibodies

## Abstract

In primary Sjögren's disease (pSjD), there is a well-documented increased risk of hematological malignancies, particularly lymphomas, whereas studies examining the incidence of solid tumors have often yielded conflicting results. Data concerning the incidence of breast and gynecological cancers are similarly inconsistent. The aim of this study was to further investigate the incidence of these so-called female cancers by assessing not only their occurrence following the diagnosis of pSjD but also prior to it. By linking the Malmö Sjögren's disease register with the Swedish National Cancer Register, 25 cases of female cancers were identified among patients with pSjD (mean follow-up time: 45.8 years), compared to 50.70 expected cases (SIR, 0.49; 95 % CI, 0.30–0.69; *P* < 0.001). The incidence of female cancers was significantly reduced both prior to (*P* < 0.001) and following (*P* < 0.001) the diagnosis of pSjD. The decrease was most evident for breast cancer (SIR, 0.46; 95 % CI, 0.24–0.69; *P* < 0.001). A reduction was also noted for ovarian cancer (SIR, 0.21; 95 % CI, 0.00–0.62; *P* < 0.001); however, the reliability of this estimate is limited by the small number of observed cases. Our findings suggest a reduced risk of female cancers, primarily driven by a decrease in breast cancer incidence, not only following but also preceding the diagnosis of pSjD. These results, combined with the observation that autoantibodies characteristic of pSjD often emerge several years before clinical onset, raise the question of whether an autoimmune component may confer a protective effect against breast cancer in pSjD.

## 1 Background

Primary Sjögren's disease (pSjD) is an autoimmune systemic disease characterized by lymphocytic infiltration of the exocrine glands, resulting in impaired secretory function. The salivary and lacrimal glands are primarily affected; however, various other organs and mucous membranes may also be involved, leading to a wide range of systemic symptoms [1,2]. In addition, patients with pSjD exhibit a well-documented and significantly increased incidence of lymphoma, particularly in the exocrine glands [[3], [4], [5]].

While numerous studies have investigated the incidence of solid malignancies diagnosed following the diagnosis of pSjD, their findings have been limited and inconsistent, generally suggesting no significant deviation from expected population rates – with the possible exception of an increased risk of thyroid cancer [[6], [7], [8]]. Notably, a recent review has drawn attention to a potentially reduced incidence of breast cancer in patients with pSjD, although this finding appears to be confined to European populations [9]. Given the strongly skewed sex distribution in pSjD, with over 90 % of patients being female [1,10], the suggestion of a decreased risk of a hormone-dependent cancer such as breast cancer is particularly noteworthy and warrants further investigation.

The etiology of pSjD remains poorly understood. However, we have demonstrated that the production of autoantibodies highly characteristic of pSjD often occurs many years prior to both the clinical onset and diagnosis of the disease. Specifically, anti–Ro60/SSA, anti–Ro52/SSA, and anti–La/SSB autoantibodies can be detected up to 18–20 years before the appearance of symptoms and the subsequent diagnosis of pSjD [11,12]. These findings suggest that pSjD-predisposing immunological mechanisms are active well before the clinical onset of the disease. If these underlying autoimmune processes are linked to, or perhaps even contribute to, the reduced incidence of breast cancer observed in European patients diagnosed with pSjD, it is plausible that this effect on breast cancer incidence may be evident even before a formal diagnosis of Sjögren's disease is made. To investigate this hypothesis, we expand upon previous studies on breast cancer in pSjD by including cases diagnosed both before and after the pSjD diagnosis, as well as by analyzing data over an extended period (1958–2019). Given that the reduced incidence of breast cancer has been attributed to lower estrogen exposure in pSjD patients [13], we also included gynecological cancers, as many of these are also hormone sensitive.

The aims of the present study were therefore twofold: to replicate previous epidemiological investigations on breast and gynecological cancers, and to assess the incidence of these cancers both prior to and following the diagnosis of pSjD in comparison with the general population. Our pSjD-specific register, which has been operational since 1984, in combination with data from the National Cancer Register, provides the dates of diagnosis for both pSjD and each cancer type, thus facilitating long-term follow-up of individual patients.

## 2 Patients and methods

### 2.1 Study design

This retrospective cohort study was based on data from the Malmö Sjögren's disease register, which was linked to the Swedish National Cancer Register and the Regional Cancer Center Register for southern Sweden. The aim was to assess whether the incidence of breast and gynecological cancers in female patients with pSjD, both before and after the diagnosis of pSjD, differed from the expected rates in the general population.

The study was conducted in accordance with the Declaration of Helsinki and was approved by the Swedish Ethical Review Authority, Stockholm (Dnr 2020–02801). Written informed consent was obtained from all participants to serve as research subjects.

### 2.2 Data sources

The Malmö Sjögren's disease register is a research database that, at the time of this study, included 490 patients diagnosed according to the American-European Consensus Group criteria [14]. Since 1984, consecutive patients with pSjD attending the Department of Rheumatology at Skåne University Hospital Malmö, Sweden, have been enrolled in the register, which contains a comprehensive collection of clinical and laboratory data. Most of the patients reside in southern Sweden, and 446 of them are women. The Swedish National Cancer Register, established in 1958, covers the entire Swedish population. It is mandatory for all healthcare providers to report newly diagnosed cancer cases to the register. In addition, there are six Regional Cancer Center registries where registration, including thorough verification, occurs before a case is entered into the national register. The National Cause of Death Register, which includes all deaths in Sweden, is updated annually with data from the National Board of Health and Welfare.

### 2.3 The study cohort

The study cohort comprised all 446 female patients included in our Sjögren's disease register. Information on cancer from 1958 to 2019 was collected from the two mentioned cancer registry data sources. Thus, the individual observation period for cancer began in 1958 or from the year of birth after 1958, and continued until the first diagnosis of female cancer, death, or the study closure date of December 31, 2019, whichever occurred first.

### 2.4 Identification of female cancers before and after pSjD diagnosis

The linkage of the Sjögren's disease register to the cancer registries was carried out using the patients' national personal identification number, which is assigned to all permanent residents of Sweden. Of the 446 female patients in the Sjögren's disease register, two patients were excluded due to the absence of a complete national personal identification number, which prevented matching with the national registries. The remaining 444 pSjD patients were linked to the National Cancer Register and the Regional Cancer Center Register to obtain data on breast and gynecological cancer. The following types of cancer were recorded, with the corresponding codes according to the International Classification of Diseases, 7th Revision (ICD7): 170 for breast cancer, 171 for cervical cancer, 172 and 174 for uterine cancer, 175 for ovarian cancer, 173 for placenta, and 176 for other and unspecified female genital organs. Linkage to the National Cause of Death Register provided information regarding the patients' date of death, if applicable. The cohort's follow-up time for cancer incidence was calculated using the time period 1969–2019 and amounted to 20,332 person-years, with a mean follow-up time of 45.8 years (standard deviation [SD] 8.44). The earliest cancer case found by us was from 1969. Considering reported indications of a limited data quality during the national register's early years [15], and in order not to overestimate the number of person-years, we decided to omit the time period 1958–1968 during the calculation of person-years (but to keep it in the calculations of cancer incidence and observation period for cancer).

### 2.5 Statistical analysis

Descriptive statistics were used to summarize demographic and clinical characteristics. Standardized incidence ratios (SIRs) with 95 % confidence intervals (CIs) were calculated by comparing observed cancer cases in the pSjD cohort with expected cases in the general population of women from the same geographical region. The total incidence of female cancer was calculated, as well as incidence observed both before and after the pSjD diagnosis, respectively. The number of cases was weighted by the number of follow-up years, and adjustments were also made for age (in 10-year intervals) and calendar year. Fisher's exact test was used to assess differences in categorical variables and clinical markers. R (version 4.1.2; R Foundation for Statistical Computing, Vienna, Austria) was used to calculate SIRs with 95 % CIs. All statistical analyses were conducted using IBM SPSS Statistics, version 25 (IBM Corp., Armonk, NY, USA). A two-sided *P*-value of <0.05 was considered statistically significant.

## 3 Results

A total of 25 cases of breast or gynecological cancer (referred to hereafter as ‘female cancer’) were identified among the 444 pSjD patients, whereas approximately twice that number (50.70) was expected, resulting in a significantly reduced SIR of 0.49 (95 % CI 0.30–0.69, *P* < 0.001) (Table 1). The most common type of female cancer among the pSjD patients was breast cancer, accounting for 16 of the 25 total cases. This was compared with an expected number of 34.52, resulting in a decreased SIR of 0.46 (95 % CI 0.24–0.69, *P* < 0.001). The incidence of ovarian cancer was also significantly lower, with only one observed case compared to 4.80 expected cases, yielding an SIR of 0.21 (95 % CI 0.00–0.62, *P* < 0.001). Six cases of uterine cancer and two cases of cervical cancer were observed, both showing a trend toward lower incidence, with SIRs of 0.84 and 0.72, respectively. No cases of more rare forms of female cancer were observed among the pSjD patients during the follow-up period. A significantly lower incidence of female cancer was found both before (n = 10) and after (n = 15) the pSjD diagnosis, with a tendency for a more pronounced reduction prior to the pSjD diagnosis: SIR 0.44 (*P* < 0.001) and SIR 0.54 (*P* < 0.001), respectively (Table 1).

**Table 1:** Incidence of female cancers in patients with pSjD.

Type of female cancer	Observed (*n*)	Expected (*n*)	SIR	95 % Cl	*P value*
Breast	16	34.52	0.46	0.24–0.69	<0.001
Uterine	6	7.15	0.84	0.17–1.51	0.639
Cervical	2	2.76	0.72	0.00–1.73	0.588
Ovarian	1	4.80	0.21	0.00–0.62	<0.001
Other types	0	1.35	no data	no data	no data

Total, before pSjD diagnosis	10	22.84	0.44	0.16–0.71	<0.001
Total, after pSjD diagnosis	15	27.85	0.54	0.27–0.81	<0.001
Total	25	50.70	0.49	0.30–0.69	<0.001

The observed number of female cancer cases is based on 444 female pSjD patients, and the expected number of cases is based on data from the general female population of the same geographic region. The number of cases was weighted by the duration of follow-up, with adjustments made for age and calendar year. A two-sided *P*-value of <0.05 was considered statistically significant.

pSjD, primary Sjögren's disease; *n*, number; SIR, standardized incidence ratio; CI, confidence interval.

Data on age and autoantibodies in the pSjD patients are presented in Table 2, with autoantibody data obtained at the time of pSjD diagnosis. Patients with female cancer were older at the time of pSjD diagnosis compared to those without female cancer (61.1 ± 10.9 years vs. 53.4 ± 15.2 years, *P* = 0.002). Similarly, among the pSjD patients still alive at the time of the register study in 2020, those with breast cancer were older (73.8 years, SD 12.3, n = 8) compared to those without breast cancer (66 years, SD 15.2, n = 304) (results not shown).

**Table 2:** Data on age and autoantibodies in pSjD patients.

	No female cancer	Breast cancer	Breast cancer before pSjD diagnosis	Breast cancer after pSjD diagnosis	Female cancer, total
Number of pSjD patients	419	16	6	10	25
Age at pSjD-diagnosis, mean (SD)	53.4 (15.2)	63.2 (8.7)	64.7 (7.8)	62.3 (9.5)	61.1 (10.9)
Age at cancer diagnosis, mean (SD)	–	64.4 (15.1)	51.3 (9.0)	72.2 (12.4)	62.4 (15.0)
ANA positive, %	72.0	71.4	66.7	75.0	73.9
Anti-SSA positive, anti-SSB negative, %	18.8	31.3	16.7	40.0	28.0
Anti-SSA negative, anti-SSB positive, %	0.2	0.0	0.0	0.0	0.0
Anti-SSA positive, anti-SSB positive, %	44.8	31.3	50.0	20.0	36.0
RF positive, %	59.1	40.0	40.0	40.0	50.0

The analyses are based on 444 female pSjD patients. Data on ANA were unavailable for 24 patients, data on anti-SSA and/or anti-SSB were unavailable for 4 patients, and data on RF were unavailable for 24 patients.

pSjD, primary Sjögren's disease; ANA, antinuclear antibody; RF, rheumatoid factor.

Results for anti-SSA and anti-SSB autoantibodies were available for all 25 patients with female cancer and for 415 of the 419 patients without female cancer. Although no statistically significant difference was observed (*P* > 0.05), it is noteworthy that only 5 (31.3 %) of the 16 patients with breast cancer were positive for both anti-SSA and anti-SSB, compared to 44.8 % among those without female cancer, and that it was even lower (20 %) among the subgroup diagnosed with breast cancer after the pSjD diagnosis.

Table 3 presents the autoantibody characteristics of the 16 patients with breast cancer, including the number of years between the pSjD and cancer diagnoses. Cancer occurred up to 26 years before and after the pSjD diagnosis. There was no clear temporal clustering of breast cancer around the time of pSjD diagnosis, although five patients were diagnosed with breast cancer within five years of their pSjD diagnosis. Of the 16 patients, all but one had symptoms of dry mouth, and all but three had symptoms of dry eyes, at pSjD diagnosis. Salivary gland biopsy had been performed in 13 patients, of whom 11 had focal lymphocytic sialadenitis (Supplementary information: Table S1).

**Table 3:** Characteristics of each of the 16 pSjD patients with breast cancer.

	Breast cancer before pSjD diagnosis						Breast cancer after pSjD diagnosis									
Patient number^a^	**1**	**2**	**3**	**4**	**5**	**6**	**7**	**8**	**9**	**10**	**11**	**12**	**13**	**14**	**15**	**16**
Years between pSjD and cancer	26	22	10	9	8	5	2	2	2	4	8	11	12	14	18	26
Age at pSjD diagnosis	67	68	65	76	58	54	72	58	51	63	74	47	60	75	66	57
ANA	–	+	+	+	–	+	NA	+	+	–	+	+	–	NA	+	+
anti-SSA	+	–	–	+	+	+	+	+	–	–	+	–	–	+	+	+
anti-SSB	+	–	–	+	–	+	–	+	–	–	–	–	–	–	–	+
RF	+	–	–	+	–	NA	–	+	–	–	–	–	+	+	–	+

pSjD, primary Sjögren's disease; NA, data not available; ANA, antinuclear antibody; RF, rheumatoid factor.

a Each patient is assigned a number (1−16) indicating the number of years between pSjD diagnosis and breast cancer diagnosis.

## 4 Discussion

Previous studies on female cancer in pSjD have primarily focused on malignancies diagnosed after the pSjD diagnosis. As a result, limited attention has been given to cancer incidence preceding disease onset. Our study addresses this gap by being one of the few to report data on female cancers diagnosed both before and after the diagnosis of pSjD. In our cohort – consisting of patients who fulfilled the American-European Consensus Group criteria for pSjD – we observed a significantly reduced overall risk of female cancer, largely attributable to a 50 % reduction in breast cancer incidence.

This finding is consistent with some earlier studies. Using data from the French hospitalization database, Goulabchand et al. [16] compared hospitalized patients with pSjD to matched hospitalized controls and found a lower incidence of breast cancer in the pSjD group, even after adjusting for potential confounders such as low socioeconomic status, hypertension, diabetes, and obesity (aHR 0.60 [0.49–0.74]). A decreased overall incidence of female cancer was also noted among pSjD patients in that study. Our findings are further supported by a study from Hemminki et al. [17], based on the Swedish Hospital Discharge Register, which reported a reduced incidence of breast cancer across 33 autoimmune diseases, with the most pronounced reduction observed in patients with Sjögren's disease (SIR 0.46 [0.26–0.75]). In addition, a nationwide population-based cohort study from Taiwan found a lower risk of Sjögren's disease among female breast cancer patients compared to women without breast cancer (aHR 0.21 [0.09–0.48]) [18].

However, several other studies on pSjD have not found evidence of a reduced incidence of breast cancer [6,[19], [20], [21]], and a few have even reported a trend toward increased risk [22,23]. The cancer incidence rates reported in these latter studies should be interpreted with some caution due to limitations such as relatively few patients, considerable heterogeneity in inclusion and exclusion criteria and for pSjD diagnosis. For example, a study from Taiwan that reported an elevated risk of breast cancer utilized insurance claims data from the National Health Insurance Research Database, which may have led to over-diagnosis of pSjD cases [23]. Nevertheless, a recent meta-analysis suggests the existence of geographical variation, with a reduced risk of breast cancer observed among pSjD patients in European populations, whereas studies from the United States, Asia, and Argentina did not report similar findings [9].

Both breast and ovarian cancers are more prevalent in Western countries, and many of the risk factors associated with ovarian cancer overlap with those for breast cancer [24,25]. In this context, our finding of a reduced risk of ovarian cancer among pSjD patients is particularly noteworthy and represents a novel observation. Hemminki et al. [17] reported a decreased risk of ovarian cancer in a large study based on the Swedish Hospital Discharge Register – including patients with 33 different autoimmune diseases – but not specifically for pSjD patients. By contrast, a recent study from Turkey by Aslan et al. [26] found an increased risk of ovarian cancer among pSjD patients; however, that study had some limitations: it used alternative diagnostic criteria for pSjD and did not adjust for geographic region in the statistical analysis. Other studies from France, Taiwan, and Spain have not identified any association between pSjD and ovarian cancer risk [16,19,20]. Although our findings suggest a decreased risk, only a single case of ovarian cancer was identified in our pSjD cohort. The resulting SIR should therefore be interpreted with caution due to limited statistical precision, and confirmation in larger studies is needed.

Compared to pSjD patients without female cancer, those with female cancer were, in our study, older both at the time of pSjD diagnosis (Table 2) and, in the case of breast cancer, also at the time of the present study. These age differences are expected, as cancer – particularly breast cancer – is more prevalent in older women. For example, a woman diagnosed with pSjD at the age of 30 and followed in the register for 20 years would, by the age of 50, still have a relatively low baseline risk of cancer. In contrast, a woman diagnosed with pSjD later in life and currently aged 80 would naturally have had a higher cumulative risk of developing cancer.

It is well established that breast cancer risk increases with cumulative estrogen exposure, both endogenous and through hormone replacement therapy [27]. Women with pSjD have been reported to have lower cumulative estrogen exposure compared to sicca controls without pSjD [13], which may be one factor contributing to the lower breast cancer incidence observed in pSjD patients. However, one of the previous studies that reported a decreased breast cancer risk adjusted for key estrogen-related reproductive factors – namely, number of children and age at first childbirth – thereby partly challenging the notion that estrogen exposure alone explains the association [17]. A reduced prevalence of traditional breast cancer risk factors – such as overweight, low physical activity, and alcohol consumption – may also contribute to the decreased incidence observed in our pSjD cohort. However, as data on these variables were not available, no firm conclusions can be drawn. Beyond lifestyle-related factors, it is important to consider that pSjD itself may exert a protective effect against breast cancer, potentially through mechanisms related to immune surveillance, as proposed by Goulabchand et al. [16] and Witkowski Durand Viel et al. [28].

We have previously demonstrated that autoantibodies characteristic of pSjD, including anti-SSA and anti-SSB, often emerge many years – up to two decades – before clinical onset and diagnosis of the disease [11,12]. This prolonged preclinical autoimmune activity, together with our current finding of a reduced risk of female cancer not only after but also before the pSjD diagnosis, is consistent with the hypothesis that a pre-existing autoimmune response may confer partial protection against breast cancer, potentially mediated by specific autoantibodies or immune mechanisms active already during the pre-symptomatic phase of pSjD. The potential association between autoantibodies and cancer risk has been explored in several other autoimmune diseases. In patients with systemic lupus erythematosus (SLE), a lower risk of certain female cancers, including breast cancer, has been reported [29]. Mechanistic studies suggest that this may be related to cell-penetrating anti-dsDNA antibodies, which could exert anti-cancer effects in cells with DNA repair defects [30]. Moreover, it has been shown that positivity for multiple (three or more) SLE autoantibodies is associated with a significantly reduced risk of breast cancer [31]. Similarly, in a recent study of patients with systemic sclerosis (scleroderma), data suggest that cancer risk may vary according to autoantibody subsets, with a decreased risk observed in patients positive for multiple scleroderma-related autoantibodies [32].

In the present study, we did not specifically investigate whether the risk of female cancers in pSjD patients varied by autoantibody subtype. However, it is noteworthy that only 5 (31.3 %) of the 16 breast cancer patients tested positive for both anti-SSA and anti-SSB, and that this fraction was even lower (20.0 %) among the 10 patients diagnosed with breast cancer after the pSjD diagnosis. Given the small number of patients, this observation should be interpreted with caution. Further investigation is needed in larger cohorts to explore whether the association between autoantibodies and cancer risk in pSjD parallels the findings observed in SLE and systemic sclerosis.

As discussed earlier, the primary limitations of the present study include the lack of data on hormonal factors influencing female cancer risk in pSjD, such as age at first childbirth, age at menopause, and other established breast cancer risk factors, including body mass index, alcohol consumption, and smoking. Additionally, information on whether the patients had received immunosuppressive or immunomodulatory treatments was unavailable. The study also lacks data on specific breast cancer characteristics, such as hormone receptor status, which may further limit our understanding of the observed cancer risk reduction. Another limitation is the relatively small number of pSjD patients with cancer in our cohort. In particular, the low incidence of cervical and ovarian cancers may result in potentially unstable SIR estimates. It may be argued that the accuracy of our cancer incidence figures might have benefitted from taking into account the concept of immortal time bias; i.e., the fact that some cancer individuals having a pSjD-predisposing type of autoimmune reactivity died before they were diagnosed with pSjD and thus included in the pSjD cohort. However, we decided not to perform such a calculation because of the ambiguity of identifying the pre-diagnostic time point at which an individual should qualify to be labelled as a pSjD patient *in spe*.

Strengths of the current study includes the use of a large outpatient pSjD register of well characterized patients, established in 1984. This cohort is likely to represent a broader spectrum of pSjD patients compared to hospitalization-based studies, which typically include patients with more severe disease. Furthermore, the long follow-up period for cancer diagnoses and the high reliability of the Swedish National Cancer Register [33], which has been operational since 1958, enhance the credibility and robustness of our findings.

## 5 Conclusions

This study demonstrates a significantly reduced risk of female cancers, particularly breast cancer, among patients with pSjD. Our analysis, which includes cancer diagnoses both before and after the pSjD diagnosis, indicates that this reduced risk is present not only after the diagnosis but also several years before the disease clinically manifests. The results support the hypothesis that immunological mechanisms active long before the pSjD diagnosis may influence cancer development, potentially through a protective autoimmune effect, particularly with respect to breast cancer.

Despite these findings, several limitations exist in the study, including the lack of data on hormonal and other traditional risk factors for breast cancer, as well as the relatively small cohort of cancer diagnoses. Further research is needed to confirm these results in larger pSjD cohorts and to investigate the potential role of autoantibodies, such as anti-SSA and anti-SSB, in reducing the risk of breast cancer.

## CRediT authorship contribution statement

**Bitte Sjöström:** Conceptualization, Formal analysis, Investigation, Methodology, Writing – original draft, Writing – review & editing. **Anders Bredberg:** Conceptualization, Formal analysis, Methodology, Writing – original draft, Writing – review & editing. **Peter Olsson:** Methodology, Resources, Writing – review & editing. **Susann Ullén:** Formal analysis, Methodology, Writing – review & editing. **Johannes H. van der Stoep:** Methodology, Writing – review & editing. **Gunnel Henriksson:** Conceptualization, Formal analysis, Funding acquisition, Investigation, Methodology, Writing – original draft, Writing – review & editing.

## Funding

This study was supported by the Region Skane Research Fund [grant number 2022-1544]. The funder has not been involved in any aspect of the study and writing of the report.

## Declaration of competing interest

The authors declare that they have no known competing financial interests or personal relationships that could have appeared to influence the work reported in this paper.

## Data Availability

The data underlying this article are available in the article and in its online supplementary information.
