# Supplementary material for: Female cancer incidence before and after diagnosis of primary Sjögren's disease: A retrospective cohort study
Source: J Transl Autoimmun. 2026 Jan 21;12:100352. doi: 10.1016/j.jtauto.2026.100352 (PMC12874099; doi:10.1016/j.jtauto.2026.100352)
Supplement: Multimedia component 1 [file mmc1.docx]

|  | Breast cancer **before** pSjD diagnosis | | | | | | Breast cancer **after** pSjD diagnosis | | | | | | | | | |
| --- | --- | --- | --- | --- | --- | --- | --- | --- | --- | --- | --- | --- | --- | --- | --- | --- |
| Patient number* | **1** | **2** | **3** | **4** | **5** | **6** | **7** | **8** | **9** | **10** | **11** | **12** | **13** | **14** | **15** | **16** |
| Years between pSjD and cancer | 26 | 22 | 10 | 9 | 8 | 5 | 2 | 2 | 2 | 4 | 8 | 11 | 12 | 14 | 18 | 26 |
| Age at pSjD diagnosis | 67 | 68 | 65 | 76 | 58 | 54 | 72 | 58 | 51 | 63 | 74 | 47 | 60 | 75 | 66 | 57 |
| ANA | - | + | + | + | - | + | NA | + | + | - | + | + | - | NA | + | + |
| anti-SSA | + | - | - | + | + | + | + | + | - | - | + | - | - | + | + | + |
| anti-SSB | + | - | - | + | - | + | - | + | - | - | - | - | - | - | - | + |
| RF | + | - | - | + | - | NA | - | + | - | - | - | - | + | + | - | + |
| Dry mouth (symptom) | y | y | y | y | y | n | y | y | y | y | y | y | y | y | y | y |
| Dry eyes (symptom) | y | y | y | y | y | n | n | y | y | n | y | y | y | y | y | y |
| Labial salivary gland biopsy | NA | + | + | NA | + | + | - | + | + | + | - | + | + | + | + | NA |
| Schirmer's test | + | + | + | + | - | - | + | + | + | + | + | + | + | NA | + | + |
| UWS | - | + | + | + | NA | + | + | + | + | + | + | + | + | + | + | + |
| C3 (g/L) | 0.96 | 1.05 | 0.91 | NA | 0.65 | 1.03 | 0.70 | 1.46 | 0.88 | 1.35 | NA | 1.00 | 1.29 | NA | 0.84 | NA |
| C4 (g/L) | 0.22 | 0.13 | 0.39 | NA | 0.25 | 0.27 | 0.10 | 0.20 | 0.20 | 0.55 | NA | 1.15 | 0.20 | NA | 0.36 | NA |

**Supplementary Table S1**

Characteristics of each of the 16 pSjD patients with breast cancer

*Each patient is assigned a number (1-16) indicating the number of years between pSjD diagnosis and breast cancer diagnosis.

pSjD, primary Sjögren’s disease; y, yes; n, no; NA, data not available; ANA, antinuclear antibody; RF, rheumatoid factor; UWS, unstimulated whole saliva flow rate (sialometry).
